# 5-Bromo-1-methyl­indolin-2-one

**DOI:** 10.1107/S1600536809021564

**Published:** 2009-06-13

**Authors:** Mao-Sen Yuan, Qi Shuai, Lin Wang, Xiao-Zhou Li, Rui-Jin Yu

**Affiliations:** a College of Science, Northwest A&F University, Yangling 712100, Shanxi Province, People’s Republic of China

## Abstract

The title mol­ecule, C_9_H_8_BrNO, approximates a full planar conformation. The inter­planar angle between the benzene and five-membered rings of the indoline system is 1.38 (1)°. There is an obvious π-delocalization involving the N—C=O group in the five-membered ring, which is greater than that involving the N—C C(benzene) group.

## Related literature

For the biological activity of indole-2-one derivatives, see: Frohner *et al.* (2005 ▶); Xie *et al.* (2007 ▶). For a related structure, see: Lipkowski *et al.* (1995 ▶).
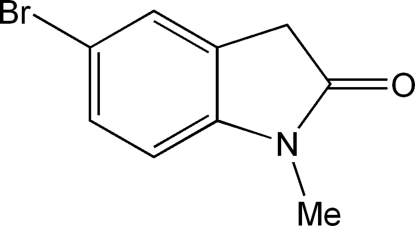


## Experimental

### 

#### Crystal data

C_9_H_8_BrNO
*M* *_r_* = 226.07
Monoclinic, 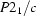

*a* = 10.5134 (4) Å
*b* = 11.0926 (4) Å
*c* = 7.7168 (3) Å
β = 103.229 (2)°
*V* = 876.06 (6) Å^3^
*Z* = 4
Mo *K*α radiation
μ = 4.64 mm^−1^
*T* = 293 K
0.47 × 0.45 × 0.44 mm

#### Data collection

Bruker APEXII CCD area-detector diffractometer
Absorption correction: multi-scan (*SADABS*; Bruker, 2005 ▶) *T*_min_ = 0.122, *T*_max_ = 0.130
6684 measured reflections
2012 independent reflections
1655 reflections with *I* > 2σ(*I*)
*R*_int_ = 0.022

#### Refinement

*R*[*F* ^2^ > 2σ(*F* ^2^)] = 0.028
*wR*(*F* ^2^) = 0.071
*S* = 1.06
2012 reflections
111 parameters
H-atom parameters constrained
Δρ_max_ = 0.37 e Å^−3^
Δρ_min_ = −0.49 e Å^−3^

### 

Data collection: *APEX2* (Bruker, 2005 ▶); cell refinement: *SAINT* (Bruker, 2005 ▶); data reduction: *SAINT*; program(s) used to solve structure: *SIR97* (Altomare *et al.*, 1999 ▶); program(s) used to refine structure: *SHELXL97* (Sheldrick, 2008 ▶); molecular graphics: *SHELXTL* (Sheldrick, 2008 ▶); software used to prepare material for publication: *WinGX* (Farrugia, 1999 ▶).

## Supplementary Material

Crystal structure: contains datablocks global, I. DOI: 10.1107/S1600536809021564/bh2231sup1.cif

Structure factors: contains datablocks I. DOI: 10.1107/S1600536809021564/bh2231Isup2.hkl

Additional supplementary materials:  crystallographic information; 3D view; checkCIF report

## Figures and Tables

**Fig. 1.**
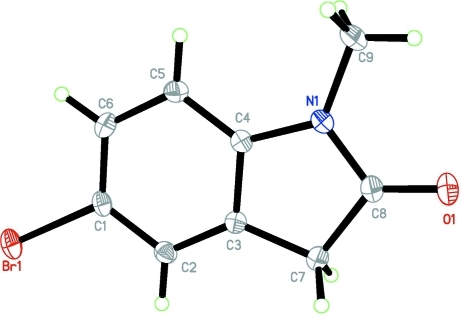
The molecular structure of (I). Displacement ellipsoids are drawn at the 30% probability level.

**Fig. 2.**
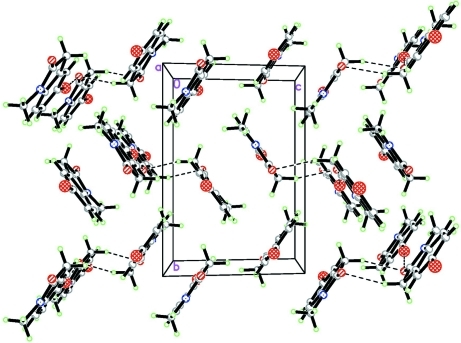
A a axis view of the molecular packing of (I).

## supplementary crystallographic information

### Comment

Indole-2-one derivatives have been widely explored due to their wide range of biological activities (Frohner *et al.*, 2005; Xie *et al.*, 2007). Indole-2-one may also be used as a precuesor for synthesizing organic lighting compounds because of its perfect planar conformation. In the course of exploring new luminescent compounds, we obtained an intermediate compound 5-bromo-1-methylindolin-2-one (I). Here we report the structure and synthesis of (I).

The molecule lies approximately in a plane (Fig. 1). The interplanar angle between the benzene group and the five-membered ring is 1.38 (1)° and the maximum displacement from the least-squares plane defined by all the 9 atoms of the indoline framework is 0.057 (3) Å for atom C9. The alternating long and short bond lengths are observed in the benzene ring: C1—C2 = 1.386 (3), C2—C3 = 1.370 (3), C3—C4 = 1.398 (3), C4—C5 = 1.377 (3), C5—C6 = 1.393 (3), C1—C6 = 1.375 (4) Å. The difference among the three C—N bond lengths is obvious, C4—N1 = 1.394 (3), C8—N1 = 1.376 (3), C9—N1 = 1.451 (3) Å, and indicates the presence of an appropriate π delocalization involving the C8—N1 and C8—O1 bonds. The structural conformation of the title molecule (I) is similar to that of 1-methylindolin-2-one (Lipkowski *et al.*, 1995).

The molecules are packed in *P*2_1_/*c* space group which is different from that of 1-methylindolin-2-one (*Pbca*). There are no classic hydrogen bonds in this structure. However, the weak intermolecular interaction C7—H7B···O1 (2 - *x*, -*y*, 1 - *z*), is helpful to the stabilization of the packing (Fig. 2). This intermolecular hydrogen bond is characterized by the bond lengths of 0.97 (C7—H7B) and 2.51 Å (H7B···O1).

### Experimental

1-Methylindolin-2-one (0.50 g) was dissolved in acetonitrile (10 ml). After cooling the mixture to 263 K, an acetonitrile solution of NBS (0.60 g) was slowly added. After stirring for 24 h., the mixture was poured into ice water and further stirred for 1 h. The solution was extracted with chloroform and dried over Na_2_SO_4_. After removing the solvent, the crude product was purified by recrystallization from ethanol, affording the title compound, (I) (0.58 g, 76%). Then the compound (I) was dissolved in a mixture of solvents, dichloromethane and isopropyl ether, and pink block crystals were formed on slow evaporation at room temperature over one week.

### Refinement

All H atoms were placed in geometrically calculated positions and refined using a riding model with C—H = 0.93 (aromatic CH), 0.97 (CH_2_ groups) or 0.96 Å (CH_3_ group). Their isotropic displacement parameters were set to 1.2 times (1.5 times for the methyl group) the equivalent displacement parameter of their parent atoms.

### Crystal data

**Table:**

C_9_H_8_BrNO	*F*(000) = 448
*M**_r_* = 226.07	*D*_x_ = 1.714 Mg m^−^^3^
Monoclinic, *P*2_1_/*c*	Mo *K*α radiation, λ = 0.71073 Å
Hall symbol: -P 2ybc	Cell parameters from 2899 reflections
*a* = 10.5134 (4) Å	θ = 2.7–27.5°
*b* = 11.0926 (4) Å	µ = 4.64 mm^−^^1^
*c* = 7.7168 (3) Å	*T* = 293 K
β = 103.229 (2)°	Block, pink
*V* = 876.06 (6) Å^3^	0.47 × 0.45 × 0.44 mm
*Z* = 4	

### Data collection

**Table:**

Bruker APEXII CCD area-detector diffractometer	2012 independent reflections
Radiation source: fine-focus sealed tube	1655 reflections with *I* > 2σ(*I*)
graphite	*R*_int_ = 0.022
φ and ω scans	θ_max_ = 27.5°, θ_min_ = 2.0°
Absorption correction: multi-scan (*SADABS*; Bruker, 2005)	*h* = −13→10
*T*_min_ = 0.122, *T*_max_ = 0.130	*k* = −11→14
6684 measured reflections	*l* = −10→10

### Refinement

**Table:**

Refinement on *F*^2^	Secondary atom site location: difference Fourier map
Least-squares matrix: full	Hydrogen site location: inferred from neighbouring sites
*R*[*F*^2^ > 2σ(*F*^2^)] = 0.028	H-atom parameters constrained
*wR*(*F*^2^) = 0.071	*w* = 1/[σ^2^(*F*_o_^2^) + (0.03*P*)^2^ + 0.5204*P*] where *P* = (*F*_o_^2^ + 2*F*_c_^2^)/3
*S* = 1.06	(Δ/σ)_max_ < 0.001
2012 reflections	Δρ_max_ = 0.37 e Å^−^^3^
111 parameters	Δρ_min_ = −0.49 e Å^−^^3^
0 restraints	Extinction correction: *SHELXL97* (Sheldrick, 2008), Fc^*^=kFc[1+0.001xFc^2^λ^3^/sin(2θ)]^-1/4^
0 constraints	Extinction coefficient: 0.0099 (11)
Primary atom site location: structure-invariant direct methods	

### Fractional atomic coordinates and isotropic or equivalent isotropic displacement parameters (Å^2^)

**Table:**

	*x*	*y*	*z*	*U*_iso_*/*U*_eq_	
Br1	0.28547 (2)	0.07936 (3)	0.20946 (4)	0.04945 (13)	
C1	0.4602 (2)	0.0991 (2)	0.1860 (3)	0.0355 (5)	
C2	0.5537 (2)	0.0170 (2)	0.2694 (3)	0.0374 (5)	
H2	0.5316	−0.0460	0.3366	0.045*	
C3	0.6793 (2)	0.0308 (2)	0.2506 (3)	0.0329 (5)	
C4	0.7111 (2)	0.1265 (2)	0.1505 (3)	0.0316 (5)	
C5	0.6183 (2)	0.2080 (2)	0.0666 (3)	0.0400 (6)	
H5	0.6401	0.2710	−0.0006	0.048*	
C6	0.4907 (2)	0.1926 (2)	0.0858 (3)	0.0399 (6)	
H6	0.4258	0.2460	0.0306	0.048*	
C7	0.8012 (3)	−0.0418 (2)	0.3173 (4)	0.0435 (6)	
H7A	0.7903	−0.1238	0.2725	0.052*	
H7B	0.8244	−0.0438	0.4464	0.052*	
C8	0.9037 (2)	0.0246 (2)	0.2444 (3)	0.0404 (6)	
C9	0.9086 (3)	0.2039 (3)	0.0514 (4)	0.0537 (7)	
H9A	0.9993	0.1827	0.0715	0.081*	
H9B	0.9006	0.2850	0.0908	0.081*	
H9C	0.8688	0.1978	−0.0733	0.081*	
N1	0.84354 (19)	0.12225 (19)	0.1502 (3)	0.0360 (4)	
O1	1.01834 (18)	−0.0009 (2)	0.2635 (3)	0.0580 (5)	

### Atomic displacement parameters (Å^2^)

**Table:**

	*U*^11^	*U*^22^	*U*^33^	*U*^12^	*U*^13^	*U*^23^
Br1	0.03020 (16)	0.0645 (2)	0.05570 (18)	−0.00283 (13)	0.01405 (11)	−0.01295 (13)
C1	0.0260 (11)	0.0432 (15)	0.0375 (11)	−0.0013 (10)	0.0077 (9)	−0.0084 (10)
C2	0.0390 (13)	0.0348 (13)	0.0409 (12)	−0.0030 (11)	0.0142 (10)	0.0002 (10)
C3	0.0326 (12)	0.0315 (12)	0.0343 (11)	−0.0007 (10)	0.0068 (9)	−0.0015 (9)
C4	0.0315 (12)	0.0321 (12)	0.0313 (11)	−0.0028 (10)	0.0076 (9)	−0.0044 (9)
C5	0.0439 (14)	0.0339 (14)	0.0441 (13)	−0.0008 (11)	0.0136 (11)	0.0049 (10)
C6	0.0354 (13)	0.0387 (14)	0.0425 (13)	0.0073 (11)	0.0027 (10)	−0.0004 (11)
C7	0.0353 (14)	0.0452 (15)	0.0499 (14)	0.0056 (11)	0.0098 (11)	0.0097 (12)
C8	0.0338 (14)	0.0463 (15)	0.0415 (13)	0.0003 (11)	0.0093 (10)	−0.0034 (11)
C9	0.0457 (16)	0.0552 (18)	0.0667 (18)	−0.0071 (13)	0.0263 (14)	0.0092 (14)
N1	0.0329 (11)	0.0388 (11)	0.0382 (10)	−0.0035 (9)	0.0121 (8)	0.0001 (9)
O1	0.0322 (11)	0.0747 (15)	0.0686 (12)	0.0084 (10)	0.0147 (9)	0.0060 (11)

### Geometric parameters (Å, °)

**Table:**

Br1—C1	1.900 (2)	C6—H6	0.9300
C1—C6	1.375 (4)	C7—C8	1.515 (4)
C1—C2	1.386 (3)	C7—H7A	0.9700
C2—C3	1.370 (3)	C7—H7B	0.9700
C2—H2	0.9300	C8—O1	1.214 (3)
C3—C4	1.398 (3)	C8—N1	1.376 (3)
C3—C7	1.502 (3)	C9—N1	1.451 (3)
C4—C5	1.377 (3)	C9—H9A	0.9600
C4—N1	1.394 (3)	C9—H9B	0.9600
C5—C6	1.393 (3)	C9—H9C	0.9600
C5—H5	0.9300		
			
C6—C1—C2	121.7 (2)	C3—C7—C8	103.6 (2)
C6—C1—Br1	119.70 (18)	C3—C7—H7A	111.0
C2—C1—Br1	118.56 (18)	C8—C7—H7A	111.0
C3—C2—C1	118.4 (2)	C3—C7—H7B	111.0
C3—C2—H2	120.8	C8—C7—H7B	111.0
C1—C2—H2	120.8	H7A—C7—H7B	109.0
C2—C3—C4	120.1 (2)	O1—C8—N1	124.9 (2)
C2—C3—C7	132.2 (2)	O1—C8—C7	127.8 (3)
C4—C3—C7	107.7 (2)	N1—C8—C7	107.4 (2)
C5—C4—N1	128.4 (2)	N1—C9—H9A	109.5
C5—C4—C3	121.7 (2)	N1—C9—H9B	109.5
N1—C4—C3	109.9 (2)	H9A—C9—H9B	109.5
C4—C5—C6	117.7 (2)	N1—C9—H9C	109.5
C4—C5—H5	121.1	H9A—C9—H9C	109.5
C6—C5—H5	121.1	H9B—C9—H9C	109.5
C1—C6—C5	120.4 (2)	C8—N1—C4	111.38 (19)
C1—C6—H6	119.8	C8—N1—C9	123.7 (2)
C5—C6—H6	119.8	C4—N1—C9	124.8 (2)
			
C6—C1—C2—C3	0.1 (4)	C2—C3—C7—C8	179.2 (3)
Br1—C1—C2—C3	179.31 (18)	C4—C3—C7—C8	0.4 (3)
C1—C2—C3—C4	0.6 (3)	C3—C7—C8—O1	−179.9 (3)
C1—C2—C3—C7	−178.2 (2)	C3—C7—C8—N1	0.4 (3)
C2—C3—C4—C5	−0.9 (4)	O1—C8—N1—C4	179.3 (2)
C7—C3—C4—C5	178.1 (2)	C7—C8—N1—C4	−1.0 (3)
C2—C3—C4—N1	180.0 (2)	O1—C8—N1—C9	4.0 (4)
C7—C3—C4—N1	−1.0 (3)	C7—C8—N1—C9	−176.2 (2)
N1—C4—C5—C6	179.5 (2)	C5—C4—N1—C8	−177.7 (2)
C3—C4—C5—C6	0.5 (3)	C3—C4—N1—C8	1.3 (3)
C2—C1—C6—C5	−0.5 (4)	C5—C4—N1—C9	−2.6 (4)
Br1—C1—C6—C5	−179.67 (18)	C3—C4—N1—C9	176.5 (2)
C4—C5—C6—C1	0.2 (4)		

## References

[bb1] Altomare, A., Burla, M. C., Camalli, M., Cascarano, G. L., Giacovazzo, C., Guagliardi, A., Moliterni, A. G. G., Polidori, G. & Spagna, R. (1999). *J. Appl. Cryst.* **32**, 115–119.

[bb2] Bruker (2005). *APEX2*, *SAINT* and *SADABS* Bruker AXS Inc., Madison, Wisconsin, USA.

[bb3] Farrugia, L. J. (1999). *J. Appl. Cryst.* **32**, 837–838.

[bb4] Frohner, W., Monse, B., Braxmeier, T. M., Casiraghi, L., Sahagun, H. & Seneci, P. (2005). *Org. Lett.* **7**, 4573–4576. 10.1021/ol051550a 16209482

[bb5] Lipkowski, J., Luboradzki, R., Stefaniak, L. & Wojcik, J. (1995). *J. Chem. Crystallogr.* **25**, 299–308.

[bb6] Sheldrick, G. M. (2008). *Acta Cryst.* A**64**, 112–122. 10.1107/S0108767307043930 18156677

[bb7] Xie, J., Sun, J., Zhang, G., Houghten, R. A. & Yu, Y. (2007). *J. Comb. Chem.* **9**, 566–568. 10.1021/cc070010x 17497930

